# Superior vena cava stenosis due to lipomatosis of the right atrium

**DOI:** 10.1111/jocs.13548

**Published:** 2018-03-11

**Authors:** Masashi Yokose, Hirotaka Sato, Hirohiko Akutsu, Yoshio Misawa

**Affiliations:** ^1^ Division of Cardiovascular Surgery Jichi Medical University Tochigi Japan

1

Lipomatosis of the heart is usually found in the right atrium (RA), occasionally in the wall of the myocardium, and may result in arrhythmias.^1^, ^2^ We present images of lipomatosis of the RA resulting in superior vena cava (SVC) stenosis.

A 77‐year‐old female presented with paroxysmal palpitations found to be frequent premature atrial contractions (PAC) on Holter monitoring. Her physical examination was unremarkable. A transthoracic echocardiogram revealed multiple RA masses involving the interatrial septum ranging in size from 1 to 6 cm (Figure 1A). A computed tomography (CT) scan showed that the masses compressed the RA‐SVC border resulting in SVC stenosis (Figure 1B). Magnetic resonance imaging revealed that the masses had high signal intensity on T1 imaging consistent with a lipid lesion. At the time of surgery, the central venous pressure was 8 mmHg. A median sterntomy was performed, and an arterial cannula was placed in the ascending aorta and venous cannulae were placed in the SVC and the right femoral vein. Following cardioplegic arrest, a right oblique atriotomy was performed and multiple lipomatous masses were resected from the interatrial septum. A small rim of tissue was left in place adjacent to the atrioventricular and sinus nodes. The septal defect and RA incision were closed primarily with a running suture. The patient tolerated the procedure well. The histology of the mass was consistent with benign lipomatous hypertrophy (Figure 2A). A post‐operative CT scan showed enlargement of the RA cavity but a persistant stenosis of the SVC (Figure 2B). A dilated azyous vein was noted which served as a collateral from the SVC (Figure 2C). The patient is now 15 months post‐op, has only an occasional PAC, and has no evidence of an SVC syndrome.

**Figure 1:**
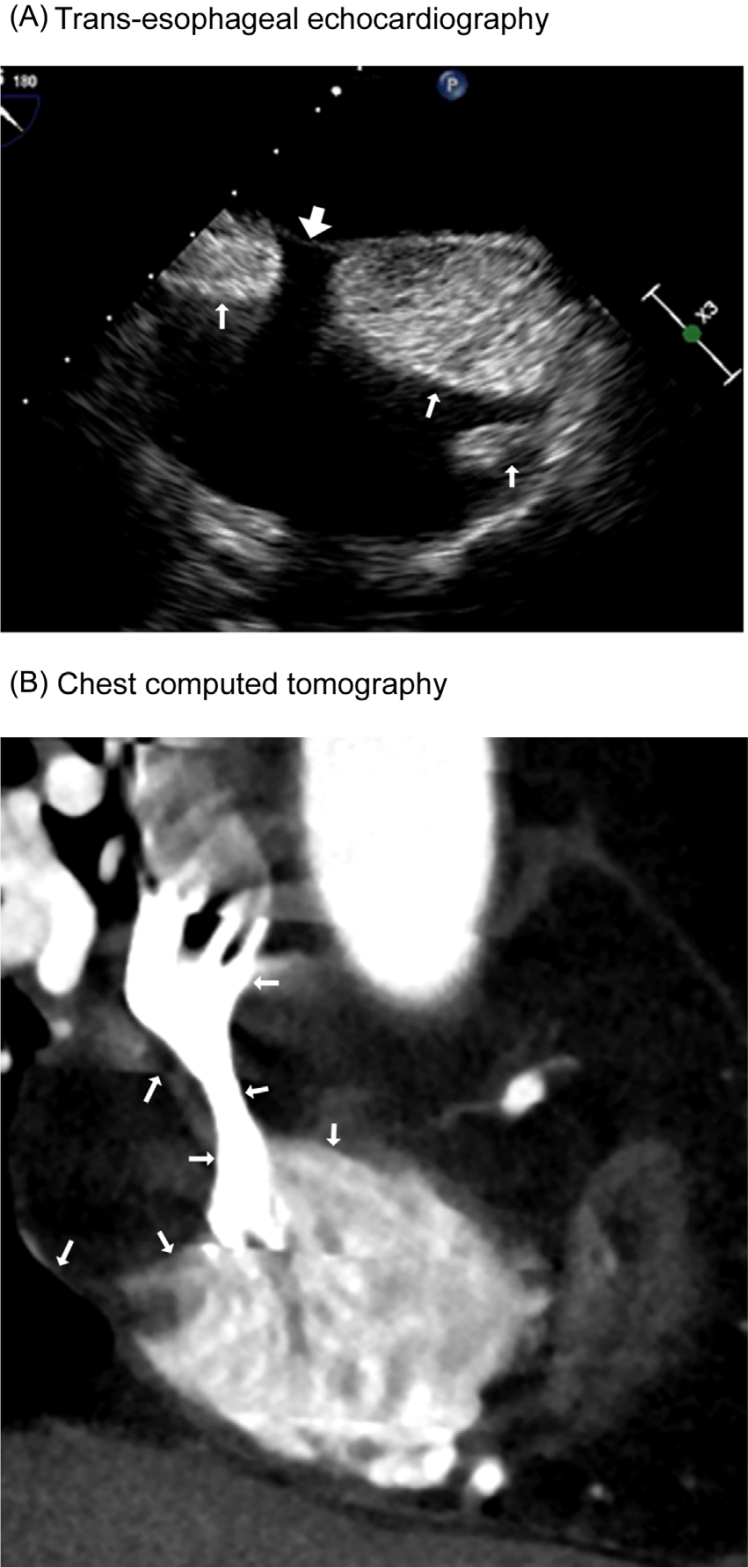
Preoperative findings. A, Trans‐esophageal echocardiographic findings showing multiple masses located in the right atrium. The right atrial cavity is narrowed by the masses (thin arrows). Masses at the interatrial septum locate sparing the foramen ovale (broad arrow). B, Chest computed tomography showing huge masses in the right atrium and narrowing of the superior vena cava are observed (arrows)

**Figure 2:**
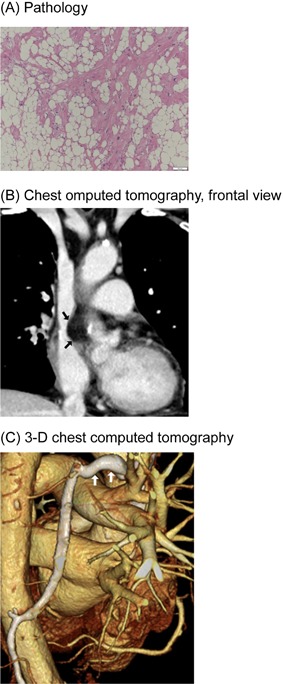
Post‐operative findings. A, Pathologic findings of the resected masses (Hematoxylin‐Eosin stain). Tumor consists of lipomatous hypertrophy with benign adipocyte proliferation. Fat cells with no fibrous capsule proliferate. B, Chest computed tomography shows expansion of the right atrial cavity with the remaining stricture of the superior vena cava caused by the residual mass (black arrows). C, The azygos vein (white arrows) becomes dilated serving a collateral vein of the superior vena cava

## CONFLICTS OF INTEREST

None declared.
